# Phytotoxicity and Identification of Active Compounds from *Elaeocarpus floribundus* Blume Plant for Controlling Weeds

**DOI:** 10.1155/2024/4995447

**Published:** 2024-08-19

**Authors:** Kawsar Hossen, Toshiaki Teruya, Shunya Tojo, Hisashi Kato-Noguchi

**Affiliations:** ^1^ Department of Agriculture Faculty of Science Noakhali Science and Technology University, Noakhali 3814, Bangladesh; ^2^ Faculty of Education University of the Ryukyus, 1 Senbaru, Nishihara, Okinawa 903-0213, Japan; ^3^ Graduate School of Engineering and Science University of the Ryukyus, 1 Senbaru, Nishihara, Okinawa 903-0213, Japan; ^4^ Department of Applied Biological Science Faculty of Agriculture Kagawa University, Miki 761-0795, Kagawa, Japan; ^5^ The United Graduate School of Agricultural Sciences Ehime University, Matsuyama 790-8566, Japan

## Abstract

Phytotoxic compounds isolated and identified from different plants have the ability to use as plant-based herbicides. Phytotoxic chemicals may be essential to weed management and environmental protection in order to reduce the indiscriminate use of synthetic pesticides. It has been reported that *Elaeocarpus floribundus* plant possesses phytotoxic compounds. The leaf extracts of this species demonstrated significant growth inhibition against the tested plants (dicot plant lettuce and plant monocot timothy) and inhibition was dose- and species-dependent pattern. Two phytotoxic compounds were separated using different purifications methods and identified as compounds **1** and **2**. All phytotoxic compounds displayed potent growth limitation against the tested species (cress). The compound concentrations needed for the inhibition of 50% growth (IC_50_ value) of tested species ranged from 1.06 to 8.53 *µ*M (micromolar). Findings of this research suggest that these compounds might be responsible for the phytotoxicity of *Elaeocarpus floribundus* plant. The results of this study may be helpful for the development of natural herbicide to control weeds.

## 1. Introduction

In the past few years, with increasing of the globalization and international trade, dissemination of the invasive weeds has increased, posing a severe menace to the agricultural output and environmental ecosystems [1]. Changes in the climate are partially responsible for the spread of exotic plant species over the world and the breakdown of traditional community and social structures [2]. The IPBES (International Plan for Biodiversity and Ecosystem Services) 2020 report stated that invasive weeds are considered a significant factor in the degradation of biodiversity and the cause of serious health problems for humans and animals [3, 4]. The species composition and variety of different species are altered by spreading of various weeds. Different weeds also reduce the productivity and effectiveness of the invaded areas by displacing native species quickly [5–7]. Lower crop net output worldwide may be caused by a number of issues, including low agricultural seed quality, the application of traditional and incorrect sowing techniques, inappropriate planting times with inadequate doses, insufficient input use, inadequate irrigation, and ineffective or noneffective weed control methods. However, most farmers ignore weed infestations that can directly impede crop development and significantly reduce grain and straw yields [8]. Naturally occurring competition between weeds and crops for space, moisture, nutrients, and light results in a considerable decrease in agricultural yield [9, 10]. Weed plants might also serve as a host to numerous insects and pathogens that harm different crop species. A study found that weed infestation caused a 13.2% yearly loss in crop yield globally, which is equal to thousand million people's annual provisions supply [11]. Some characteristics associated with weeds include long-lasting seeds, a rapid ability to germinate, a quick growth habit, the ability to survive in unfavorable environments, and the lack of a requirement for specific environmental conditions for emergence. These characteristics help the weed make a harmful obstacle for crop production. Hence, weed management is essential or performs a pivotal role for crop protection and food supply.

There are some traditional weed management strategies such as manual, mechanical, chemical, and biological [12–14]. Manual weed control methods are the best way to manage weeds in a crop field, but the cost and manpower requirements are very high to make it practical for widespread crop cultivation. Chemical control of weed is a very popular and common weed control strategy, mainly depending on the application of synthetic herbicides. The indiscriminate use of synthetic herbicides or chemicals has led to several issues, such as environmental pollution, residues of synthetic herbicides, and weed resistance. In affluent nations, agriculture primarily relies on synthetic herbicides to manage weeds. However, since the 1980s, herbicides without a novel MOA have been launched (mechanism of action) [15–17]. The intensive use of synthetic herbicides without novel mechanisms of action has led to the evolution of resistance in 513 biotypes of 267 weed species against 21 different herbicides worldwide, across several cropping systems [18]. The USA (United States of America) has the majority of herbicide resistance cases, with over 160 plant species affected. Heavy herbicide use also has several additional negative effects, including increased chemical prices, the risk of groundwater contamination from runoff and leaching, and issues with irrigation water recycling [19]. Concerns among the general public about herbicides' effects on the environment and public health are also growing.

The necessity to create an eco-friendly, long-lasting tool or instrument for weed control has arisen due to the development of synthetic herbicide-resistant weeds, stemming from the absence of new synthetic herbicides with novel modes of action and increased awareness among people about the effects of herbicides [20]. Biological weed control is one of the important methods to control weed through the application of allelopathic compounds (allelopathy) which are isolated from natural sources mostly from different plants [21]. Biological weeding strategy on the basis of allelopathy is considered one of the best weed control methods [22]. The term “allelopathy” refers to a well-known biological method involving the secretion of allelochemicals (secondary metabolites) by plants or the decomposition of plant products discharged into the soil and environment [23, 24]. Different allelochemicals such as phenolic substances, fatty acids, terpenes, sterols, alkaloids, essential oils, and saponins are necessary for a plant's interaction with its environment but are not essential for the development and growth of the plant [25]. In the context of a natural ecosystem or environment, allelopathy happens when allelochemicals generated by one plant are dispersed onto nearby plants in similar population. The allelochemicals impact their development, germination, survival, and reproduction. Several allelopathic compounds that have been identified from different plants have been investigated in relation to their potential use in weed management.

Most of them already proved their harmful activities against weed species by suppressing germination, shoot, and root growth and also hampering water, nutrients uptake, and photosynthesis [26–28]. Natural substances with allelopathic properties often have a short half-life, so there is no need to worry about residues in the soil. Various studies claim that the natural substances or allelochemicals of particular plant species could play a vital role in weed management tactics due to this benefit as well as their effectiveness [29, 30]. The application of extracts from various plant species, rich in natural compounds that have phytotoxic effects against weeds, might reduce the use of synthetic herbicides. Additionally, it could protect humans from health hazards and save the environment from pollution [31]. Thus, the use of allelochemicals for controlling weeds may be of great importance in reducing the indiscriminate application of synthetic herbicides.

The tree *Elaeocarpus floribundus* Blume, locally known as jalpai in Bangladesh, is one of the most common medicinal species in Bangladesh under the Elaeocarpaceae family. This species is a nondeciduous tree, generally cultivated in the Asian regions of East and South Asia, such as India and Bangladesh. The plant species are grown in different areas of the world such as Australia, China, Fiji, Madagascar, Hawaii, Japan, Mauritius, Thailand, and Malaysia [32, 33]. This tree is found in the eastern Himalayan region at heights of over 900 meters, in the northern region of Kanara's nondeciduous forest, and in the western part of coastal areas. In India, *Elaeocarpus floribundus* is found in different regions such as Jalpaiguri, Alipurduar, the northern parts, and Bihar. It is believed that Jalpaiguri is a district of India originated from the words “jalpai,” which means “the plant of jalpai,” and “guri,” which means “plant trunk” [34]. This tree is an underutilized and unexploited fruit plant that grows in homestead areas with little to no maintenance. The size and shape of the fruit are similar to olive fruit; hence, it is named Indian olive or jalpai in Bangla. *Elaeocarpus floribundus* is a medium- to large-sized tree that bears simple, alternate leaves. Sometimes, some of the leaves turn red or orange, which is a very important feature of this tree. The flowers of this tree start to appear in the months of April to May, and the fruits mature from August to October. The fruit is a drupe, greenish in color, with the edible portion, the mesocarp, found around the seed. Both immature and mature fruits have a sourish flavor and are commonly used to prepare pickles and chutney. The bark and leaves are typically used for mouthwash, and the fruits also possess antiseptic properties. The fruits have the ability to decrease and fix the activities of Ag (silver) biosynthesis, displaying a growth inhibition effect against both Gram-positive and Gram-negative bacteria [35]. This tree wood has different usages including plywood, fiberboard, constructions, and furniture. Many parts of this plant, including the roots, barks, leaves, and fruits, are traditionally used to treat a variety of diseases.

The plant has several pharmaceutical properties those are used for the treatment of various disorders like arthritis, high pressure, diabetes, and dysentery [36]. Besides, this plant is also very famous for possessing different biological effects including antiaging, anticancer, antitumor, antiseptic, antioxidant, and antibacterial [37, 38]. There have been many studies on the plant's various therapeutic applications and other uses, and we expect that it may contain allelopathic properties as well as allelopathic compounds. Therefore, current research was carried out to isolate and characterize phytotoxic compounds by using several isolation steps from *Elaeocarpus floribundus* for controlling weeds.

## 2. Materials and Methods

### 2.1. Research Plant Species

The *Elaeocarpus floribundus* species leaves were assembled from several regions of NSTU (Noakhali Science and Technology University), Bangladesh (22° 47′ 31″N and 91° 06′ 07″E) in the months of April and May, 2019. The collected leaf samples were cleaned under running water for removing debris, dirt, and different contaminants. The cleaned leaves were placed in a shady location for drying. After desiccation, these leaves were powdered using a blender. The leaf powder was then stored in a polybag and placed in the refrigerator before use. Two plant species, lettuce (*Lactuca sativa* L.) (a monocot species) and timothy (*Phleum pratense* L.) (a dicot species), were selected for determining phytotoxic activity through a bioassay experiment.

### 2.2. Extraction Procedure and Growth Assay

The extraction bioassay study was carried out to determine phytotoxic activity of *Elaeocarpus floribundus* and developed an exact isolation technique: 60 g leaves were extracted using 380 mL methanol (70% aqueous MeOH) for the time of 48 h and filtered by a filter paper (No. 2, 125 mm; Toyo Ltd., Tokyo, Japan). The remaining residues were once again submerged in an equal amount of methanol for 24 hours and then refined. The extracted residues were combined and dried using a rotavapor at 40°C. The dried leaf residues were dissolved in 140 mL of methanol to prepare different assay concentrations, such as 0.001, 0.003, 0.01, 0.03, 0.1, and 0.3 g dry weight equivalent extract per milliliter. These concentrations (6) were then applied onto a filter paper (No. 2, 28 mm; Toyo Roshi Ltd., Tokyo, Japan) in 28 mm Petri dishes. After the extracted residues were dried, the Petri dishes were wetted using 0.6 mL of a 0.05% aqueous solution of polyoxyethylene sorbitan monolaurate (Tween 20, Nacalai Tesque, Inc., Kyoto, Japan). Ten lettuce seeds (of uniform size) and ten emerged timothy seeds were placed on each Petri dish. The control treatment consisted only of Tween 20 without *Elaeocarpus floribundus*. Finally, the Petri dishes were placed in a germinator for 48 hours at a temperature of 25°C in dark conditions, and the growth of the seedlings was assessed.

### 2.3. Isolation of the Active Compounds

**Figure 1:**
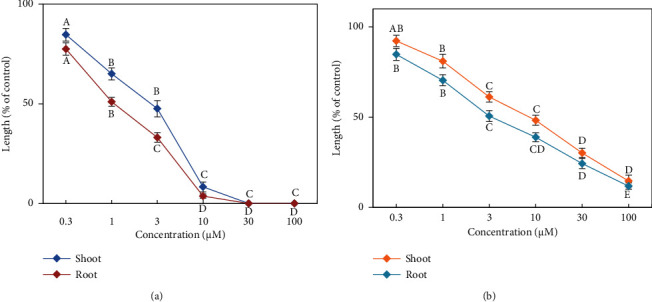
Phytotoxic activity by the compounds **1** (a) and **2** (b) on cress seedlings growth at different concentrations. Significant differences are denoted by different letters at 5% level of probability.

Isolation and purification of active phytotoxic compounds are briefly narrated in our preceding research [39]. The compounds 1 and 2 were separated from the silica gel column using a solvent mixture of 70% ethyl acetate (EtOAc) in hexane (n-hexane), from the Sephadex LH-20 column using a 40% aqueous methanol solution, and from the C18 cartridge (reverse-phase) using a 40% aqueous methanol solution. Subsequently, they were purified using reverse-phase high-performance liquid chromatography (HPLC) on a 4.6 × 250 mm Inertsil® ODS-3 column with a particle size of 5 *µ*m, provided by GL Science Inc., Tokyo, Japan. The HPLC was conducted at a flow rate of 0.8 mL/min with a 25% aqueous methanol solution. The chromatograms were detected at a wavelength of 220 nm, with an oven temperature of 40°C and retention times of 29–34 and 47–63 minutes, respectively. Lastly, these two active substances (Compound 1 and Compound 2) (Figure 1) were identified using high-resolution electrospray ionization mass spectrometry (HRESIMS) (performed on a Thermo Scientific Orbitrap Exploris 240 Mass Spectrometer) and proton nuclear magnetic resonance (1H NMR) (Bruker Corporation, Billerica, MA, USA).

### 2.4. Bioassay of the Characterized Compounds

Two characterized substances 1 and 2 were dissolved in 5 mL methanol to prepare the different bioassay concentrations: 0.3, 1, 3, 10, 30, and 100 *µ*M. These six concentrations were then applied onto the filter paper (No. 2, 28 mm; Toyo Roshi Ltd., Tokyo, Japan) in Petri dishes (28 mm). After the filter paper was completely dry, it was wetted using 0.6 mL of a 0.05% aqueous solution of polyoxyethylene sorbitan monolaurate (Tween 20, Nacalai Tesque, Inc., Kyoto, Japan). Ten homogenous seeds of cress and ten pre-emergence seeds of barnyard grass were placed on each Petri dish and kept in a growth chamber under constant darkness at 25°C temperature for 48 hours. The shoot and root lengths of the cress and barnyard grass seedlings were recorded after 48 h of growth and compared with the control seedlings.

### 2.5. Statistical Analysis

All of the bioassay studies were conducted with CRBD (completely randomized block design), repeated thrice, and total assay studies were replicated twice. Collected data from assay were represented as the mean ± standard error (SE). Analysis of variance (ANOVA) was conducted using SPSS software (SPSS Inc., Chicago, Illinois, USA), and significant variations within different treatments and control studies were determined using Tukey's HSD test at a 5% probability level. The concentrations required for 50% growth limitation (IC_50_ value) of the tested species in the assay experiments were determined using GraphPad Prism 6.0 (GraphPad Software, Inc., La Jolla, California, USA).

## 3. Results

### 3.1. Phytotoxic Effect of *Elaeocarpus floribundus* Leaf Extracts

**Figure 2:**
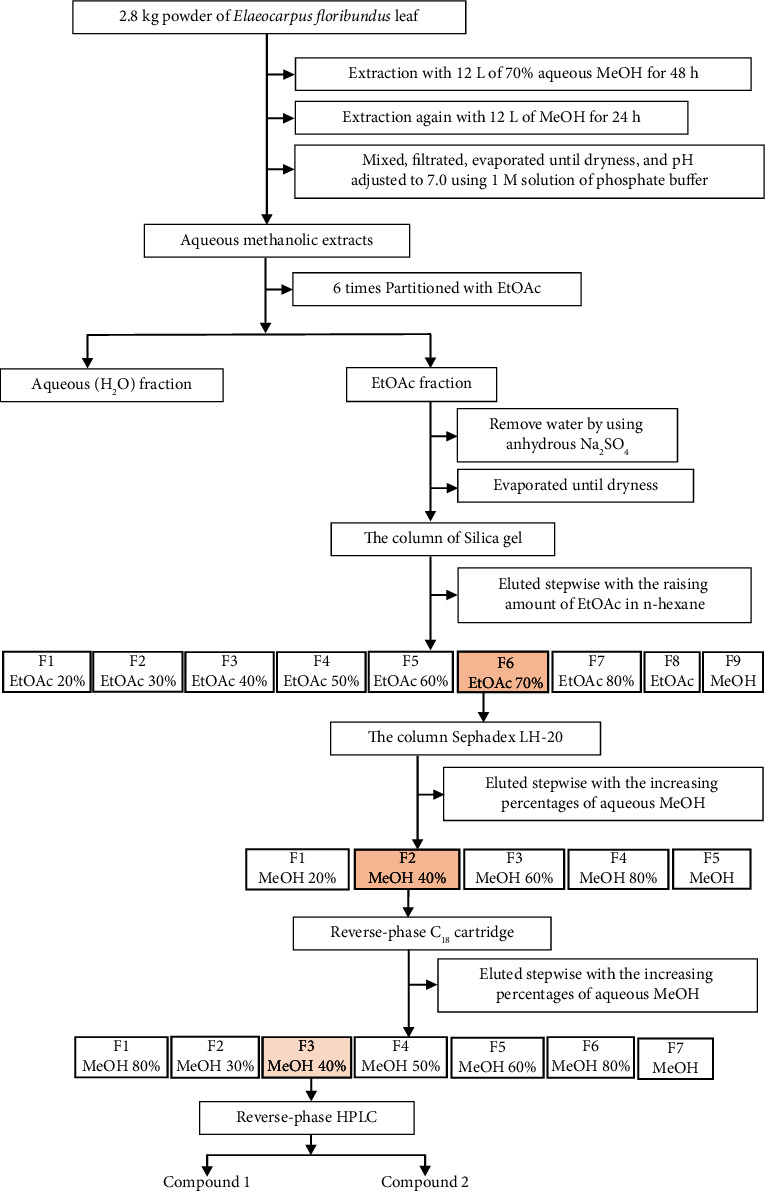
The extraction and isolation procedures of compounds **1** and **2** ((*S*)-(+)-abscisic acid and (3*R*, 6*R*, 7*E*)-3-hydroxy-4,7-megastigmadien-9-one) from the plant *Elaeocarpus floribundus*.

The leaf extracts (aqueous methanolic) of *E. floribundus* exhibited significant phytotoxicity against lettuce (*Lactuca sativa*) and timothy (*Phleum pratense*) seedling growth. The limitation of seedling growth expanded with increasing concentrations of extracts, and the growth limitation also varied within the tested species (Figure 2). In the first concentration (0.001 g dry weight equivalent *E. floribundus* extract/mL), the growth restriction was not significant, but growth suppression was significant at the greater concentrations. At a concentration of 0.03 g dry weight equivalent *E. floribundus* extract/mL, the growth of tested species' seedlings was arrested by more than 80%, except for timothy shoot growth (67.4%), compared to the growth of control seedlings. Particularly, at a concentration of 0.1 g dry weight equivalent *E. floribundus* extract/mL, the growth of lettuce seedlings was completely suppressed, while the growth of timothy shoots and root was restricted by 90.3% and 96.5%, respectively, compared to the growth of control seedlings. However, at the highest concentration (0.3 g dry weight equivalent *E. floribundus* extract/mL), the growth of seedlings from both tested species (lettuce and timothy) was completely inhibited by *E. floribundus* plant extracts. The IC_50_ values (concentrations needed for 50% growth suppression) of the tested plants ranged from 0.004 to 0.017 g dry weight equivalent *E. floribundus* extract/mL (Table 1). These IC_50_ values also indicated that root growth suppression was greater than shoot growth limitation by *E. floribundus* plant extracts.

### 3.2. Identification of Phytotoxic Compounds

**Figure 3:**
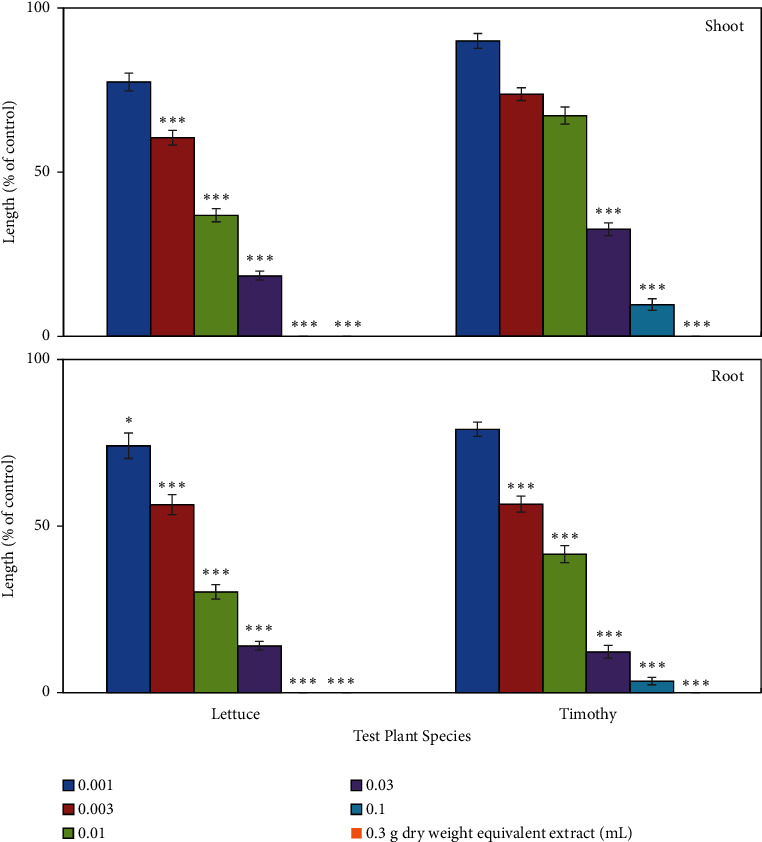
Phytotoxicity of the *Elaeocarpus floribundus* (aqueous methanolic) leaf extracts on the growth of lettuce and timothy seedlings at different concentrations. The mean ± standard error (SE) for every treatment from two experiments with three replications (seedlings (10) for every replication) for each experiment (*n* = 60) that are demonstrated. Asterisks indicated the variations between the treatments and the control: ^∗^*p* < 0.05 and ^∗∗∗^*p* < 0.001 (one-way ANOVA and LSD test by post hoc).

The phytotoxic compounds **1** and **2** (yielding 2.2 mg and 2.0 mg) were identified by comparing with the previously recorded data as (*S*)-(+)-abscisic acid [40] and (3*R*, 6*R*, 7*E*)-3-hydroxy-4,7-megastigmadien-9-one [41] and demonstrated in Figure 3 (for identification details, see the supplementary materials (available here)).

### 3.3. Phytotoxic Activity of Characterized Compounds

**Figure 4:**
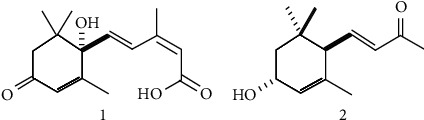
Characterized phytotoxic compounds **1** and **2,** ((*S*)-(+)-abscisic acid, and (3*R*, 6*R*, 7*E*)-3-hydroxy-4,7-megastigmadien-9-one) from the plant *Elaeocarpus floribundus*.

Two characterized compounds were tested to estimate their phytotoxic activity on the examined plant species (cress) with six concentrations (Figure 4). The results of the bioassay studies also showed that the phytotoxic activity of the characterized compounds on the examined plant growth differed significantly, and phytotoxicity increased with increasing concentrations of the identified compounds (Figure 4). The examined plant growth differed significantly at concentrations of 1 *µ*M or greater than 1 *µ*M for both compounds. At the concentrations of 30 *µ*M, species seedling growth was completely restricted by compound 1. Meanwhile, at the same concentration, seedling growth was limited to 69.83% and 75.69% (shoot and root, respectively) by compound 2 compared to control growth. The IC_50_ values of the tested plants ranged from 1.06 to 8.53 *µ*M (Table 2). Table 2 indicates that compound 1 exhibits greater phytotoxic potential than compound 2. The IC_50_ values also suggest that root growth is more sensitive than shoot growth to both compounds.

## 4. Discussion

In the preceding studies, we evaluated the allelopathic activity of the *Elaeocarpus floribundus* leaf extracts against cress (*Lepidium sativum*), alfalfa (*Medicago sativa*), barnyard grass (*Echinochloa crus-galli*), and Italian ryegrass (*Lolium multiflorum*) and found significant growth limitation [42, 43]. To support previous findings, in the present research, we have also determined the phytotoxic potentiality of *Elaeocarpus floribundus* leaf extracts against a crop plant (lettuce) and a weed plant (timothy). The aqueous methanol extract of *Elaeocarpus floribundus* leaves notably inhibited the growth of the examined plants, and the level of inhibition accelerated with increasing concentrations of the plant extracts. Many researchers also reported those kinds of growth inhibition using different plant extracts: *Senna garrettiana*, *Hyptis suaveolens*, *Schumannianthus dichotomus*, *Garcinia xanthochymus*, *Anredera cordifolia*, *Clerodendrum indicum*, *Paspalum commersonii*, *Albizia richardiana*, and *Cassia alata* [44–48]. Variations in IC_50_ value of *Elaeocarpus floribundus* leaf extracts suggested that inhibition of growth was dependent on the examined species of plant (Table 1). In various studies, it was reported that susceptibility to plant extracts differed with variation of target species, indicating differences in translocation process, mode of actions, and absorption mechanisms of allelopathic substances across plants [39]. Growth suppression potentiality of *Elaeocarpus floribundus* leaf extracts indicates that the plant may possess phytotoxic compounds.

The allelopathic activity of different plant species extracts on the recipient species is basically an outcome of the effects of phytotoxic substances that perform as toxins to inhibit the growth of recipient species or weeds [49]. It has been reported that plant extracts or allelopathic compounds affect root growth inhibition more severely than shoot growth restriction. The greater susceptibility of root growth to different plant species' extracts is due to the root being the initial organ of the plant to absorb phytotoxic compounds from plant extracts. Root tissues also exhibit more penetrability to allelopathic compounds compared to shoot tissues [50]. Allelopathic effects are normally estimated by determining the shoots' and roots' growth elongation. The results of this also demonstrated that shoots' growth was less susceptible than roots' growth to the *Elaeocarpus floribundus* plant extracts. In the previous experiments, seven allelopathic compounds such as (3*R*)-3-hydroxy-*β*-ionone, *cis*-3-hydroxy-*α*-ionone, loliolide, (+)-dehydrovomifoliol, (−)-dehydrololiolide, 5,6-epoxy-3-hydroxy-7-megastigman-9-one, and elaeocarpunone (novel compound) were identified from the *Elaeocarpus floribundus* plant extracts [42, 43]. In our current study, another two active allelopathic substances **1** and **2** were characterized from these plant extracts (Figure 3).

Compound **1** is a molecule of isoprenoid, obtained by synthesizing the carotenoid derivatives of IPP (isopentenyl diphosphate) using the pathway of MEP (methylerythritol phosphate) in the plastids [51]. It is also familiar as a plant growth regulator and plays a vital role to decrease different stresses such as transplanting shock, heat, salinity, water deficiency, and prompt crop plants establishment [52]. It enhances various physiological processes, including the closing of stomata, modulation of the root system, organization of the soil microorganism (SMO) community, triggering transcriptional and post-transcriptional gene expression, and inducing metabolic changes [53]. It has been recorded to generate reactive oxygen species (ROS) during the closing of stomata [54]. It has been demonstrated that this compound readily degrades in the soil, producing dihydrophaseic and phaseic acid. Alternatively, depending on its concentrations, this compound can either limit or stimulate the growth of roots or radicals in different plant species. Application of the compound reduces the growth rate of coleoptiles in corn. The use of this to nondormant seed showed to restrict their emergence or germination. The influence of this compound may be interlinked to its normal activity as an endogenous growth inhibitor during the formation of the seed. The growth inhibitory potentials of the compound on protoplast growth of plant species along with its significances were determined, and it demonstrated as a phytochemical in sugar beet and Beech (Fagus). It also limited the growth of protoplast of Cherry (*Prunus yedoensis*) plant at little concentrations [55]. Although this compound is a very important phytohormone, some studies showed that it has phytotoxic potential [56, 57]. This compound isolated and characterized as an allelopathic compound from several plants including *Imperata cylindrica*, *Aglaia odorata*, and *Macaranga tanarius*. The phytotoxicity of the compound has been well documented, but there is no document about the isolation, identification, and phytotoxic potential of this compound from the plant *Elaeocarpus floribundus*.

Compound **2** is a norisoprenoid type secondary metabolites, isolated from several plants such as *Artemisia myriantha*, *Golden camellias*, *Artabotrys hongkongensis*, *Artemisia mongolica*, *Malva silvestris*, *Salvinia molesta*, *Andrographis paniculate*, *Melochia umbellate*, *Tagetes erecta*, *Euphorbia humifusa*, *Vatica cinerea*, *Silene firma*, *Trigonostemon lutescens*, *Croton tiglium*, *Viburnum dilatatum*, and *Achillea alpina*. This compound has different biological activities including anticancer [58], antitumor [59], anti-HIV, antioxidant [60], and IR (insulin resistance). The phytotoxic activity of the compound has been well reported by many researchers and identified in different plant species such as *Chenopodium album*, *Cestrum parqui*, and *Tagetes* L. Although various biological properties and allelopathic potential are well established, this is the first-ever document about the identification and phytotoxicity of this compound from the *Elaeocarpus floribundus* plant.

The obtained results from the present study demonstrate that compounds **1** and **2** significantly limited the growth of cress seedling (Figure 4). The compounds I_50_ value displayed that compound **1** showed stronger allelopathic activity against cress than compound **2** (Table 2). The phytotoxic compounds affect the growth of plants through various reactions. The researchers reported that phytotoxic activity is measured by molecular structures, especially the number and position of different active groups present in the compounds [61, 62]. Thus, these compounds are liable for allelopathic potential of this plant species. Therefore, phytotoxic effects of *Elaeocarpus floribundus* leaves may provide useful information for controlling weeds as well as help develop natural herbicides.

## 5. Conclusions

The leaf extracts of *Elaeocarpus floribundus* significantly inhibited the growth of seedlings of the tested species (lettuce and timothy), and the level of suppression differed with the concentration of extracts and the species tested. Two phytotoxic substances were separated from *Elaeocarpus floribundus* leaf extracts using several purification methods and characterized by spectral analysis, identified as substances 1 and 2. All the compounds significantly inhibited the seedlings' growth of tested species cress. The results obtained from this research indicate that these two compounds (**1** and **2**) are liable for phytotoxic activity of the *Elaeocarpus floribundus* plant. Therefore, *Elaeocarpus floribundus* species might be helpful for controlling weeds in an environment friendly way. To confirm the obtained results, we will conduct this research in field condition in the future.

## Figures and Tables

**Table 1:** The IC_50_ values (concentrations needed for 50% growth suppression) of lettuce and timothy seedling by aqueous methanolic extracts of *Elaeocarpus floribundus* plant.

Test plant species		IC_50_ values (g dry weight equivalent extract/mL)	
		Shoot	Root
Dicotyledonous	Lettuce	0.005	0.004
Monocotyledonous	Timothy	0.017	0.005

**Table 2:** IC_50_ value (the compound concentrations needed for the inhibition of 50% growth) of tested plant seedlings by the compounds (**1** and **2**) from the extracts of *Elaeocarpus floribundus* leaf.

Test plant species		Compound 1	Compound 2
		(*µ*M)	
Cress	Shoot	2.57	1.06
	Root	8.53	3.18

## Data Availability

The data sets used and/or analysed during the current study are available from the corresponding author on reasonable request.
