# Supplementary material for: Phytotoxicity and Identification of Active Compounds from Elaeocarpus floribundus Blume Plant for Controlling Weeds
Source: ScientificWorldJournal. 2024 Aug 19;2024:4995447. doi: 10.1155/2024/4995447 (PMC11347024; doi:10.1155/2024/4995447)
Supplement: Supplementary Materials — Supplementary data to this article can be found online along with the article. [file 4995447.f1.docx]

**Supplementary materials**

(*S*)-(+)-abscisic acid (Compound **1**)

The molecular formula of (*S*)-(+)-abscisic acid was found to be C_15_H_20_O_4_ by HRESIMS. The ^1^H spectrum of (*S*)-(+)-abscisic acid as measured in CDCl_3_ showed the presence of four methyl proton signals at *δ*_H_ 2.04 (3H, s), 1.92 (3H, s), 1.11 (3H, s) and 1.03 (3H, s), four olefinic proton signals at *δ*_H_ 7.81 (1H, d, ­*J*=16.1 Hz), 6.17 (1H, d, ­*J*=16.1 Hz), 5.97 (1H, s) and 5.77 (1H, s) and two methylene proton signals at *δ*_H_ 2.49 (1H, d, *J*=17.1 Hz) and 2.29 (1H, d, *J*=17.1 Hz). The ^1^H NMR spectrum of (*S*)-(+)-abscisic acid was in agreement with reported data.

**Spectral data**

The optical rotation was measured on a JASCO P-1010 polarimeter. All NMR spectroscopic data were recorded on a Bruker AVANCE Ⅲ 500 MHz NMR spectrometer. Chemical shifts were reported relative to the residual solvent signal (CDCl_3_: *δ*_H_ 7.26) and (CD_3_OD: *δ*_H_ 3.31). HRESIMS were performed on a Thermo Scientific Orbitrap Exploris 240 Mass Spectrometers.

(*S*)-(+)-abscisic acid; colorless oil; [*α*] = +739.8 (*c* 0.10, EtOH) (lit.,^1^ [*α*] = +414.0); ^1^H NMR (500 MHz, CDCl_3_) *δ*_H_ 7.81 (1H, d, ­*J*=16.1 Hz), 6.17 (1H, d, ­*J*=16.1 Hz), 5.97 (1H, s), 5.77 (1H, s), 2.49 (1H, d, *J*=17.1 Hz), 2.29 (1H, d, *J*=17.1 Hz), 2.04 (3H, s), 1.92 (3H, s), 1.11 (3H, s), 1.03 (3H, s); HRESIMS *m/z* 287.1252 [M+Na]^+^ (calcd for C_15_H_20_O_4­_Na 287.1259).

(3*R*,6*R*,7*E*)-3-Hydroxy-4,7-megastigmadien-9-one (Compound 2)

(3*R*,6*R*,7*E*)-3-Hydroxy-4,7-megastigmadien-9-one

The molecular formula of (3*R*,6*R*,7*E*)-3-Hydroxy-4,7-megastigmadien-9-one was found to be C_13_H_20_O_2_ by HRESIMS. The ^1^H NMR spectrum of (3*R*,6*R*,7*E*)-3-Hydroxy-4,7-megastigmadien-9-one as measured in CDCl_3_ showed the presence of four methyl proton signals at *δ*_H_ 2.26 (3H, s),1.62 (3H, s), 1.03 (3H, s) and 0.89 (3H, s), three olefinic proton signals at *δ*_H_ 6.54 (1H, dd, *J*=15.8 Hz, 10.2 Hz), 6.10 (1H, d, *J*=15.8 Hz) and 5.63 (1H, m), two methine proton signals at *δ*_H_ 4.27 (1H, m) and 2.50 (1H, d, *J*=10.2 Hz), two methylene proton signals at *δ*_H_ 1.84 (1H, dd, *J*=13.5 Hz, 6.0 Hz) and 1.40 (1H, dd, *J*=13.5 Hz, 6.4 Hz). The ^1^H NMR spectrum of (3*R*,6*R*,7*E*)-3-Hydroxy-4,7-megastigmadien-9-one was in agreement with reported data.

**Spectral data**

The optical rotation was measured on a JASCO P-1010 polarimeter. All NMR spectroscopic data were recorded on a Bruker AVANCE Ⅲ 500 MHz NMR spectrometer. Chemical shifts were reported relative to the residual solvent signal (CDCl_3_: *δ*_H_ 7.26), (CD_3_OD: *δ*_H_ 3.31) and (Acetone-*d*6: *δ*_H_ 2.05). HRESIMS were performed on a Thermo Scientific Orbitrap Exploris 240 Mass Spectrometers.

(3*R*,6*R*,7*E*)-3-Hydroxy-4,7-megastigmadien-9-one; colorless oil; [*α*] = +136.3 (*c* 0.07, CH_2_Cl_2_) (lit.,^2^ [*α*] = +37.1); ^1^H NMR (500 MHz, CDCl_3_) *δ*_H_ 6.54 (1H, dd, *J*=15.8 Hz, 10.2 Hz), 6.10 (1H, d, *J*=15.8 Hz), 5.63 (1H, m), 4.27 (1H, m), 2.50 (1H, d, *J*=10.2 Hz), 1.84 (1H, dd, *J*=13.5 Hz, 6.0 Hz), 1.62 (3H, s), 1.40 (1H, dd, *J*=13.5 Hz, 6.4 Hz), 1.03 (3H, s), 0.89 (3H, s); HRESIMS *m/z* 209.1536 [M+H]^+^ (calcd for C_13_H_21_O_2_ 209.1542).

^1^ Smith, T. R.; Clark, A. J.; Clarkson, G. J.; Taylor, P. C.; Marsh, A. *Org. Biomol. Chem.* **2006**, *4*, 4186− 4192.

^2^ Abrosca, B. D.; DellaGreca, M.; Fiorentinoa, A.; Monaco, P.; Orianoa, P.; Temussi, F. *Phytochemistry* **2004**, *65*, 497−505.
